# Uropathogenic* Escherichia coli* in the Urine Samples of Iranian Dogs: Antimicrobial Resistance Pattern and Distribution of Antibiotic Resistance Genes

**DOI:** 10.1155/2017/4180490

**Published:** 2017-11-29

**Authors:** Amirhossein Yousefi, Saam Torkan

**Affiliations:** ^1^Faculty of Veterinary Medicine, Shahrekord Branch, Islamic Azad University, Shahrekord, Iran; ^2^Department of Small Animal Internal Medicine, Faculty of Veterinary Medicine, Shahrekord Branch, Islamic Azad University, Shahrekord, Iran

## Abstract

Resistant uropathogenic* Escherichia coli* is the most common cause of urinary tract infections in dogs. The present research was done to study the prevalence rate and antimicrobial resistance properties of UPEC strains isolated from healthy dogs and those which suffered from UTIs. Four-hundred and fifty urine samples were collected and cultured.* E. coli*-positive strains were subjected to disk diffusion and PCR methods. Two-hundred out of 450 urine samples (44.4%) were positive for* E. coli*. Prevalence of* E. coli* in healthy and infected dogs was 28% and 65%, respectively. Female had the higher prevalence of* E. coli* (*P* = 0.039). Marked seasonality was also observed (*P* = 0.024). UPEC strains had the highest levels of resistance against gentamicin (95%), ampicillin (85%), amikacin (70%), amoxicillin (65%), and sulfamethoxazole-trimethoprim (65%). We found that 21.50% of UPEC strains had simultaneously resistance against more than 10 antibiotics.* Aac(3)-IV* (77%),* CITM* (52.5%),* tetA* (46.5%), and* sul1* (40%) were the most commonly detected antibiotic resistance genes. Findings showed considerable levels of antimicrobial resistance among UPEC strains of Iranian dogs. Rapid identification of infected dogs and their treatment based on the results of disk diffusion can control the risk of UPEC strains.

## 1. Introduction

Pathogenic urinary tract infections (UTIs) occur in about 14% of dogs throughout their life [1–5]. Prevalence of UTIs in dogs had a range between 5% and 30% all around the world [1–5]. UTIs can be classified as simple uncomplicated or complex complicated infections which may spread to dangerous pathogenic diseases such as pyelonephritis, cystitis, and urethritis [1–5].

Uropathogenic* Escherichia coli *(*E. coli*) (UPEC) strains are the most significant causative agent of UTIs in both humans and dogs [1–6]. It is a Gram-negative, nonsporulating, flagellated, rod-shaped, and facultative anaerobic bacterium which belongs to Enterobacteriaceae family [7–13]. Total prevalence of UTIs caused by the UPEC strains is about 30–70% [1–6].

UTIs caused by UPEC strains are often required antibiotic therapy [1–15]. Accurate prescription of beta-lactams, aminoglycosides, quinolones, sulfonamides, tetracyclines, penicillins, and cephalosporins groups of antibiotics is effective for control and treatment of UTIs in dogs [1–5, 14, 15]. Nowadays, occurrence of antibiotic resistance is common and emerging issue in small animal medicine [1–5, 14, 15]. UPEC strains isolated from the cases of UTIs in dogs show high prevalence of resistance (85–100%) against commonly used antimicrobial agents [1–5, 14, 15]. Molecular investigations presented that the presence of certain antibiotic resistance genes including the genes that encode resistance against beta-lactams* (blaSHV*,* CITM)*, quinolones* (qnr)*, tetracycline* (tetA* and* tetB)*, trimethoprim* (dfrA1)*, gentamicin* (aac(3)-IV)*, chloramphenicol* (cat1* and* cmlA)*, sulfonamide* (sul1)*, and streptomycin* (aadA1)* is the most significant reason for occurrence of antibiotic resistance in UPEC strains [1–15].

According to the uncertain role of UPEC strains in the cases of UTIs in dogs and lack of epidemiological investigations in this field in Iran, the present research was carried out to study the prevalence rate and antimicrobial resistance properties of the UPEC strains isolated from Iranian dogs.

## 2. Materials and Methods

### 2.1. Ethical Considerations

This study was approved by the Ethical Committee of Department of Small Animal Internal Medicine of the Islamic Azad University of Shahrekord (Consent Ref Number IAUSHK 216-95).

### 2.2. Samples

From February 2015 to February 2016, a total of 200 urine samples were collected from nonmedicated adult dogs (aged >1 year) of both sexes with a presumptive diagnosis of UTIs. The inclusion criteria included clinical signs of UTIs, such as hematuria and dysuria, urinalysis results that included red blood cell counts >5 under a high-power field (HPF) and proteinuria, and >103 colony-forming units (CFU) of bacteria per milliliter of urine at the first plating. Two-hundred and fifty urine samples were also taken from healthy dogs. Urine samples were obtained by catheterization after thorough cleansing of the genital area. Urine samples were obtained from the mid-stream to minimize the outer bacterial contaminations.

### 2.3. *E. coli* Identification

Urine samples were cultured using a calibrated pipette to deliver 10 *μ*l and 100 *μ*l of samples onto Columbia agar (Merck, Germany) supplemented with 5% sheep blood and onto MacConkey agar (Merck, Germany). The blood agar plates were incubated aerobically, and the MacConkey agar plates were incubated aerobically. All samples were incubated at 37°C for 24 h until adequate growth was present. Primary plates were carefully inspected for colonies of* E. coli*, which were plated onto sheep blood agar plates; these plates were incubated at 37°C for 24 h. Suspected colonies were transferred to the Eosin Methylene Blue agar (EMB agar, Merck, Germany) plates and incubated at 37°C for 24 h. Metallic green colonies with typical* E. coli* morphologies of the EMB agar plates were identified as* E. coli* using standard techniques, including indole, Methyl Red–Voges-Proskauer (MR-VP), Triple Sugar Iron agar (TSI), and citrate biochemical testing and analysis with an API-20E system (BioMérieux, Marcy l'Etoile, France) [16].

### 2.4. DNA Extraction and* E. coli* Confirmation

Typical* E. coli* isolates were also approved using the PCR amplification of* 16S rRNA* gene. Bacterial strains were subcultured in Luria-Bertani broth (Merck, Germany) and incubated at 37°C for 24 h. Genomic DNA was extracted from growth colonies using the DNA extraction kit (Fermentas, Germany) according to manufacturer's instruction. The DNA concentration has been determined by measuring absorbance of the sample at 260 nm using spectrophotometer [17]. A PCR method was done with a total volume of 50 *μ*L including 2 mM Mgcl_2_, 1 *μ*M of forward primer (5′-AGAGTTTGATCMTGGCTCAG-3′), 1 *μ*M of reverse primer (5′-CCGTCAATTCATTTGAGTTT-3′) (Gene Bank Association Ref Number 000913.3) (919 bp), 5 *μ*L PCR buffer 10x, 200 *μ*M dNTP (Fermentas, Germany), 1 U Taq DNA polymerase (Fermentas, Germany), and 2.5 *μ*L DNA template. The DNA was then amplified by 31 successive cycles of denaturation at 95°C for 45 s, primer annealing at 59°C for 60 s, and DNA chain extension at 72°C for 60 s.

### 2.5. Antibiotic Susceptibility Testing

Antimicrobial resistance pattern of the* E. coli* isolates was studied using the simple disk diffusion technique. The Mueller–Hinton agar (Merck, Germany) medium was used for this purpose. Antibiotic resistance pattern of the* E. coli* isolates was evaluated against several types of antimicrobial agents according to the instruction of Clinical and Laboratory Standards Institute (CLSI) [18]. Seven classes of antimicrobial agents including tetracyclines (oxytetracycline (30 *μ*g/disk) and doxycycline (30 *μ*g/disk)), aminoglycosides (gentamicin (10 *μ*g/disk) and amikacin (30 u/disk)), quinolones (nalidixic acid (30 *μ*g/disk), enrofloxacin (5 *μ*g/disk), and ciprofloxacin (5 *μ*g/disk)), penicillins (amoxicillin-clavulanic acid (30 *μ*g/disk), amoxicillin (10 *μ*g/disk), and ampicillin (10 u/disk)), cephalosporins (cefazolin (30 *μ*g/disk), ceftiofur (30 *μ*g/disk), ceftazidime (30 *μ*g/disk), and cefotaxime (30 *μ*g/disk)), sulfamethoxazole-trimethoprim (25 *μ*g/disk), and chloramphenicol (30 *μ*g/disk) (Oxoid, UK) were used for this purpose. The plates containing the discs were allowed to stand for at least 30 min before being incubated at 37°C for 24 h. The diameter of the zone of inhibition produced by each antibiotic disc was measured and interpreted using the CLSI zone diameter interpretative standards [18].* E. coli* ATCC 25922 and* S. aureus* ATCC 25923 were used as quality control organism in antimicrobial susceptibility determination.

### 2.6. Detection of Antibiotic Resistance Genes

Previous method described by Momtaz et al. (2013) [6] was used for PCR amplification of the antibiotic resistance genes in the* E. coli* strains isolated from the urine samples of Iranian dogs. The DNA thermocycler (Eppendorf Mastercycler 5330, Eppendorf-Nethel-Hinz GmbH, Hamburg, Germany) was used in all PCR reactions. The PCR amplification products (15 *μ*l) were subjected to electrophoresis in a 1.5% agarose gel in 1x TBE buffer at 80 V for 30 min and stained with SYBR Green (Fermentas, Germany). All runs included a negative DNA control consisting of PCR grade water and positive DNAs of* E. coli*.

### 2.7. Statistical Analysis

Statistical analysis was performed using SPSS/21.0 software for significant relationships. Prevalence of* E. coli* strains and their antibiotic resistance properties were statistically analyzed. Statistical significance was regarded at a* P* value < 0.05.

## 3. Results and Discussion

**Figure 1:**
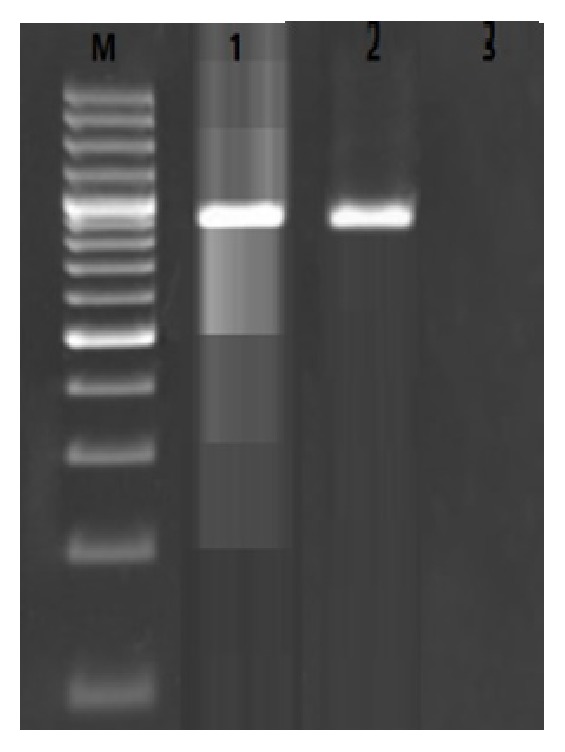
Results of the gel electrophoresis for the PCR amplification of the* 16S rRNA* gene of the* E. coli* strains isolated from Iranian dogs. M: 100 bp ladder (Fermentas, Germany), 1: positive sample for the* 16S rRNA* gene (919 bp), 2: positive control, and 3: negative control.

Table 1 shows the total prevalence of* E. coli* in the urine samples of healthy and infected dogs. We found that 200 out of 450 urine samples (44.4%) were positive for presence of* E. coli*. All of the positive isolates were also confirmed using the PCR amplification of the* 16S rRNA* gene of the* E. coli* bacteria (Figure 1). Total prevalence of* E. coli* in the urine samples taken from healthy and infected dogs was 28% and 65%, respectively. Female dogs had the higher prevalence of* E. coli*. Statistically significant differences were seen between the prevalence of* E. coli* and sex of dogs (*P* = 0.039) and also between the prevalence of* E. coli* and status of UTIs in dogs (*P* = 0.018).

Results showed that the urine samples which were collected in the summer season had the highest prevalence of* E. coli* (*P* = 0.014). Total prevalence of* E. coli* strains in the urine samples of healthy and infected dogs collected in summer and winter seasons was 50% (35/70) and 53.8% (70/130) and 8.5% (6/70) and 10% (13/130), respectively. Samples which were collected in the winter season had the lowest prevalence of bacteria. Statistically significant difference was seen between the prevalence of* E. coli* and season of sampling (*P* = 0.024).

Table 2 represents the antibiotic resistance pattern of the UPEC strains isolated from the urine samples of healthy and infected dogs.* E. coli* strains harbored the highest levels of resistance against gentamicin (95%), ampicillin (85%), amikacin (70%), amoxicillin (65%), and sulfamethoxazole-trimethoprim (65%) antibiotics.* E. coli* strains recovered from dogs which suffered from UTIs had higher prevalence of antibiotic resistance than healthy one (*P* = 0.041).* E. coli* strains recovered from male dogs had higher prevalence of antibiotic resistance than female (*P* = 0.037). Prevalence of resistance against chloramphenicol, cefazolin, and nalidixic acid was low.

All* E. coli* isolates of healthy dogs had resistance against at least one antibiotic agent (100%), while the prevalence of resistance against more than 10 antibiotic agents was 7.1%. Besides, all* E. coli* isolates of dogs which suffered from UTIs had resistance against at least 2 antibiotics (100%), while the prevalence of resistance against more than ten antibiotic agents was 21.5%.

Table 3 represents the distribution of antibiotic resistance genes among the* E. coli* strains of healthy and infected dogs. We found that* aac(3)-IV* (77%),* CITM* (52.5%),* tetA* (46.5%), and* sul1* (40%) were the most commonly detected antibiotic resistance genes among the* E. coli* isolates of Iranian dogs. Our results showed that the prevalence of* cmlA* (2.5%),* cat1* (6%), and* aadA1* (20.5%) antibiotic resistance genes was lower than other genes. Male dogs and also dogs with UTIs had a higher prevalence of antibiotic resistance genes. Statistically significant differences were seen between the prevalence of antibiotic resistance genes and sex of dogs (*P* = 0.034) and also type of urine samples (*P* = 0.043).

Results of the present investigation revealed the high prevalence of resistant strains of uropathogenic* E. coli* in the urine samples of Iranian dogs which suffered from UTIs. We found that 65% of samples taken from infected dogs were positive for* E. coli* which was considerably high. We found that healthy dogs had also a high prevalence of* E. coli*. One possible explanation for the isolation of* E. coli* from the urine samples of healthy dogs is the fact that they were sources of* E. coli* but did not show any typical clinical signs of the UTIs. Therefore, they are more dangerous than dogs which suffered from UTIs. It is because they can easily transmit the UPEC strains into the environment. This finding shows that apparently healthy dogs may be a silent source of bacteria. This finding shows that the UPEC strains can colonize into the urinary tract of dogs without any clinical sign. This is important public health problem regarding the close contact of asymptomatic dogs with human.

We also found that the female dogs had higher prevalence of UPEC strains than males in both groups. It is because female dogs have relatively short and wide urethra which causes rapid spread of infection in the upper urinary organs. Host factors such as changes in the normal vaginal and urinary flora may also put female dogs at higher risk of UTIs. Higher prevalence of UPEC strains in the urine samples of female dogs has also been reported by other researchers [3, 19–22]. Marked seasonal distribution was also seen for the prevalence of UPEC strains. UPEC strains of our investigation had the highest prevalence in the summer season. This finding is maybe due to the warmer weather of the summer which causes dehydration and reduces the volume of urination. This event can increase the chance of bacterial colonization and decrease its shedding due to the decrease in the power of flashing of urine. Besides, warmer weather makes conditions more conducive for growth of the UPEC strains.

We also found that the majority of uropathogenic* E. coli* strains were resistant to various types of antimicrobial agents. Totally, seven classes of widely used antimicrobial agents were tested. We found that UPEC strains of infected and healthy dogs harbored the highest levels of resistance against aminoglycosides, penicillins, and sulfamethoxazole-trimethoprim antibiotics. High prevalence of resistance of UPEC strains against these groups of antibiotics is partly due to the fact that all of these antibiotic groups are first-line antimicrobial agents for treatment of UTIs in dogs in Iran. First-line antibiotics are antibiotics that may be chosen empirically or based on culture and susceptibility results targeting a specific bacterium (UPEC strains in this report) with minimal impact on other bacteria. The most commonly effective antibiotics empirically used to treat first-time UTIs in dogs are potentiated cephalosporins, fluoroquinolones, sulfonamides, and chloramphenicol. They have high efficacy against Gram-positive and/or negative bacteria; however, more recently, several resistant strains have emerged, including strains resistant to penicillins (ampicillin, amoxicillin-clavulanic acid, and amoxicillin), tetracyclines (oxytetracycline and doxycycline), chloramphenicols (chloramphenicol and florfenicol), and sulfamethoxazole-trimethoprim [2, 3, 14, 19–24]. Excessive using of antimicrobial agents, including prophylactic use to prevent surgical site infections or infections associated with other urogenital diseases, may have caused the emergence of resistant strains. We found that male dogs had the higher prevalence of resistance against all tested antibiotics. This finding is maybe due to the higher levels of immunity in male dogs and also their narrow and long urethra. In addition, male dogs are in close contact with stray dogs and also polluted environments. One of the most important findings of this research is that UPEC strains isolated from asymptomatic dogs had considerable levels of resistance against several types of antibiotics (10–89%). However, the main reason for this finding is not clear but it is maybe due to the contacts of infected dogs with heathy one and transmission of resistant bacteria from infected to healthy dogs. High prevalence of resistant UPEC strains in asymptomatic dogs has a serious public health importance.

Results of the multiplex PCR method confirmed the results of disk diffusion technique. We found that majority of resistant strains of* E. coli* had the high prevalence of antibiotic resistance genes and especially* aac(3)-IV*,* CITM*, and* tetA*. It is also showed that majority of UPEC strains were resistant against more than one antibiotic agent. Unauthorized and irregular prescription of antibiotics in the field of small animals and especially dogs in Iran is the main reason for the high prevalence of antibiotic resistance.

The main mechanisms of tetracycline resistance are known to have efflux pump activity, ribosomal protection, and enzymatic inactivation. Various* tet *genes confirm resistance via these mechanisms.* TetA* (46.5%) and* tetB* (27%) genes of the UPEC strains had considerable prevalence in our study. Detection of* tetA* and* tetB* genes in tetracycline-resistant uropathogenic* E. coli *strains isolated from dogs showed the principal mechanism to be active efflux. Similar results were reported by Chang et al. (2015) [3]. They showed that of the 69 tetracycline-resistant* E. coli *isolates, the prevalence of* tetA* and* tetB* genes was 25.50% and 50.9%, respectively, which was different from our results. The main reason for the high prevalence of resistance against tetracycline in our study is the fact that this antibiotic is so cheap in Iran and veterinarians use it as a primary antibiotic for treatment of infections in dogs.

Chloramphenicol is broad-spectrum antibiotic that has rarely been used in companion animals. It was introduced as a banned antibiotic in Iran. UPEC strains of our study had a considerable resistance against chloramphenicol (15%) which showed its banned prescription. Active efflux pump* (cmlA)* and chloramphenicol acetyltransferase* (cat1)* played important roles in intrinsic and acquired chloramphenicol resistances. Overexpression of efflux pumps and acetyltransferase enzyme affecting chloramphenicol has become increasingly common in* E. coli*. We found that the prevalence of* cat1* and* cmlA* genes among the UPEC strains was 6% and 2.5%, respectively.

Several mechanisms were involved in the resistance to quinolones in* E. coli* including chromosomal genes encoding DNA gyrase and topoisomerase IV and a plasmid-mediated quinolone resistance (PMQR) including* qnr*-mediated protection of DNA from quinolone binding [24, 25]. We found that the prevalence of resistance against nalidixic acid, enrofloxacin, and ciprofloxacin was 20%, 40%, and 50%, respectively. It was also reported that all of the quinolones resistant isolates harbored the* qnr* gene. These strains were also resistant against some other types of antimicrobial agents. Therefore, additional characterization of the mechanisms of resistance to quinolones and to the other antibiotics is required.

We found that the UPEC strains had a higher level of resistance against aminopenicillins (ampicillin (85%) and amoxicillin (65%)) and a lower level of resistance against amoxicillin-clavulanic acid (25%). This result was consistent with beta-lactam antibiotics, mostly ampicillin and amoxicillin, being the most frequently used class of antimicrobial agents in dogs. The results of the study indicated that* CITM* (52.5%) and* blaSHV* (37.5%) were the most commonly *β*-lactamase antibiotic resistance genes. The* CITM* and* SHV* enzymes were the predominant plasmid-mediated *β*-lactamases found in Gram-negative Enterobacteria [26]. Thus, production of these enzymes in the UPEC strains isolated from dogs has an important concern. It can be concluded that the clinical usage of *β*-lactams in pet therapy executes a strong selective pressure in the emergence of resistant bacterial isolates.

UPEC strains of our investigation had a high prevalence of resistance against several classes of antibiotics. Presence of simultaneous resistance against several classes of antibiotics and especially sulfonamides is another important finding of our study. Sulfonamides have been used alone or in combination with trimethoprim for the treatment of UTIs in humans. However, sulfonamides have been infrequently used in dogs, because of susceptibility to adverse effects [27]. According to our study, 60% of the UPEC strains were resistant to sulfamethoxazole-trimethoprim. Resistance to sulfonamides is usually caused by the acquisition of the genes* sul1*,* sul2*, and* sul3*.* Sul1* is dihydropteroate synthase which has almost exclusively been found on large conjugative plasmids and on class 1 integrons [27]. We found that the presence of the sul1 gene was accompanied with high prevalence of* dfrA1* (dihydrofolate reductase) gene. The prevalence of* sul1* and* dfrA1* genes in the UPEC strains of our study was 40% and 35%, respectively.* DfrA1* is also associated with class 1 integrons residing in plasmids and/or the bacterial chromosome similar to sul1. Therefore, expression of one of them can facilitate expression of another one and both can facilitate resistance against sulfonamides.

Chang et al. (2015) [3] reported that the UPEC strains recovered from dogs with UTIs in Taiwan had considerable levels of resistance against gentamicin (10.50%), ampicillin (50%), amoxicillin (44.70%), amoxicillin-clavulanic acid (2.6%), chloramphenicol (31.60%), ciprofloxacin (5.30%), enrofloxacin (5.30%), nalidixic acid (38.60%), trimethoprim-sulfamethoxazole (34.20%), doxycycline (28.90%), and oxytetracycline (60.50%) antibiotics. Hagman and Greko (2003) [1] showed that the prevalence of resistance against ampicillin, chloramphenicol, enrofloxacin, gentamicin, nitrofurantoin, streptomycin, sulfamethoxazole, tetracycline, trimethoprim, and trimethoprim-sulfamethoxazole was 22.91%, 7.29%, 11.45%, 3.12%, 2.08%, 21.87%, 0%, 23.95%, 0%, and 10.41%, respectively. Idea of veterinarians to antibiotic prescription and also availability of antibiotics and their costs are the main factors which cause difference in the prevalence of antibiotic resistance in various studies.

Selection of antibiotics is depending on several factors. High urine concentrations of antimicrobials are correlated with efficacy in treatment of uncomplicated UTIs. But in complicated cases, tissue concentrations may be equally important. Most antimicrobials undergo renal elimination to a great extent, so urine concentrations may be up to 100 times peak plasma concentrations. High urine antimicrobial concentrations are important for eradication of bacteria in the urine, but for infection of the bladder wall or renal tissue it is necessary to use antimicrobials that have active concentrations in the tissues. In addition to having the appropriate antimicrobial activity and achieving effective concentrations in urine, the selected antimicrobial should be easy for owners to administer, have few adverse effects, and be relatively inexpensive [23].

Amoxicillin is more bioavailable in dogs and cats than ampicillin; hence the lower dosage achieves therapeutic concentrations in prostatic fluid [23, 28]. Clavulanic acid undergoes some hepatic metabolism and excretion, so much of the antimicrobial activity in the bladder may be due to the high concentrations of amoxicillin achieved in urine [23, 28]. Thus, despite an unfavorable susceptibility report for amoxicillin, clinically amoxicillin alone may be as effective as amoxicillin-clavulanic acid to treat UTIs [23, 28]. Ceftiofur is an injectable cephalosporin approved for respiratory disease in horses, swine, and cattle and for treatment of canine UTI caused by* E. coli*[23, 28]. Chloramphenicol has a high volume of distribution, and high tissue concentrations can be achieved, including in the prostate of male dogs and cats [23, 28]. Enrofloxacin is a member of the fluoroquinolones family approved to treat UTIs in dogs. It is concentration-dependent killers with a long postadministration effect, so once daily, high-dose therapy for a relatively short duration of treatment is effective. Fluoroquinolones should be avoided for chronic, low-dose therapy, because this encourages emergence of resistant bacteria that are cross-resistant to other antimicrobial drugs as well [23, 28]. Gentamicin and the other aminoglycosides have a low volume of distribution. The aminoglycosides have a similar spectrum of activity to that of the fluoroquinolones, but their use for UTI is limited because of the necessity of parenteral injections and the risk of toxicity with anything but short-term use. Like the fluoroquinolones, the aminoglycosides are concentration-dependent, bactericidal killers with a long postadministration effect, so once daily therapy of short duration is effective and minimizes the risk of nephrotoxicity [23, 28]. Tetracyclines are bacteriostatic, amphoteric drugs with a high volume of distribution. The tetracyclines are shed through urine without any changes, so high urinary concentrations may result in therapeutic efficacy [23, 28]. Trimethoprim is a bacteriostatic, basic drug with a high volume of distribution and a short elimination half-life, whereas the sulfonamides are bacteriostatic, acidic drugs with a medium volume of distribution and long half-lives. Microbiology services use the 1 : 20 ratio in susceptibility testing; however, the widely varying pharmacokinetic properties of this drug combination make it difficult to determine a therapeutic regimen that achieves the 1 : 20 ratio at the infection site [23, 28].

Currently, the duration of therapy for UTI is controversial. Although animals are routinely treated with antimicrobial drugs for 10–14 days, shorter duration antimicrobial regimens are routinely prescribed in human patients, including single-dose fluoroquinolone therapy. A clinical comparison of 3 days of therapy with a once daily high dose of enrofloxacin with 2 weeks of twice daily amoxicillin-clavulanic acid showed equivalence in the treatment of simple UTI in dogs. Animals with complicated UTI may require longer courses of therapy, and underlying pathology must be addressed. Chronic complicated cases of UTI, pyelonephritis, and prostatitis may require antimicrobial treatment for 4–6 weeks, with the risk of selecting for antimicrobial resistance. A follow-up urine culture should be performed after 4–7 days of therapy to determine efficacy. If the same or a different pathogen is seen, then an alternative therapy should be chosen and the culture repeated again after 4–7 days. Urine should also be cultured 7–10 days after completing antimicrobial therapy to determine whether the UTI has resolved or recurred [23, 28, 29].

In keeping with the high prevalence of UPEC strains in the UTIs, considerable prevalence of this pathogen and also many other types of pathogenic bacteria in food samples is an important risk factor for sickness, decrease of the immunity level, and occurrence of UTIs [30–49].

## 4. Conclusions

In conclusion, we identified a large number of the UPEC strains with high levels of resistance against several groups of antibiotics with respect to the high prevalence of antibiotic resistance genes. Monitoring antibiotic prescription and resistance patterns in a small animal internal medicine may serve as an early indicator of changes in the antibiotic susceptibility of clinical isolates. Using form culture-based identification, disk diffusion, and PCR-based amplification of antibiotic resistance genes provided valuable data to veterinarians for the management of persistent or recurrent UTI in dogs. Prescription of cefazolin and nalidixic acid antibiotics can be more effective for treatment of UTIs in Iranian dogs. Rapid identification of infected dogs, attentions to the results of disk diffusion method, and principles of antibiotic prescription can reduce the risk of UPEC strains in Iranian dogs.

## Figures and Tables

**Table 1:** Total distribution of uropathogenic *E. coli* strains isolated from the urine samples of Iranian dogs.

Types of samples	Number of samples collected	*E. coli* positive (%)	PCR confirmation (%)
Healthy dogs			
Male	140	30 (21.4)	30 (21.4)
Female	110	40 (36.3)	40 (36.3)
Total	250	70 (28)	70 (28)

Dogs with UTIs			
Male	120	70 (58.3)	70 (58.3)
Female	80	60 (75)	60 (75)
Total	200	130 (65)	130 (65)

*Total*	*450*	*200 (44.4)*	*200 (44.4)*

**Table 2:** Antimicrobial resistance pattern of the uropathogenic *E. coli* strains isolated from Iranian dogs.

Types of samples (number positive)	Antimicrobial resistance pattern (%)															
	Tetracyclines		Aminoglycosides		Quinolones			Penicillins			Cephalosporins				Sult	C30
	Ox^*∗*^	Deox	Gen	Amk	Nal	En	Cip	Amc	Amx	Amp	Cf	Cft	Cftz	Cfx		
Healthy dogs																
Male (30)	15 (50)	18 (60)	30 (100)	19 (63.3)	8 (26.6)	16 (53.3)	18 (60)	6 (20)	22 (73.3)	28 (93.3)	7 (23.3)	9 (30)	11 (36.6)	11 (36.6)	20 (66.6)	5 (16.6)
Female (40)	10 (25)	12 (30)	32 (80)	13 (32.5)	4 (10)	10 (25)	13 (32.5)	4 (10)	15 (37.5)	21 (52.5)	3 (7.5)	6 (15)	6 (15)	7 (17.5)	15 (37.5)	2 (5)
Total (70)	25 (35.7)	30 (42.8)	62 (88.5)	32 (45.7)	12 (17.1)	26 (37.1)	31 (44.2)	10 (14.2)	37 (52.8)	49 (70)	10 (14.2)	15 (21.4)	17 (24.2)	18 (25.7)	35 (50)	7 (10)

Dogs with UTIs																
Male (70)	43 (61.4)	36 (51.4)	70 (100)	60 (85.7)	15 (21.4)	32 (45.7)	41 (58.5)	25 (35.7)	57 (81.4)	70 (100)	18 (25.7)	20 (28.5)	20 (28.5)	27 (38.5)	50 (71.4)	13 (18.5)
Female (60)	32 (53.3)	24 (40)	58 (96.6)	48 (80)	13 (21.6)	22 (36.6)	28 (46.6)	15 (25)	36 (60)	51 (85)	12 (20)	15 (25)	13 (21.6)	15 (25)	35 (58.3)	10 (16.6)
Total (130)	75 (57.6)	60 (46.1)	128 (98.4)	108 (83.0)	28 (21.5)	54 (41.5)	69 (53.0)	40 (30.7)	93 (71.5)	121 (93.0)	30 (23.0)	35 (26.9)	33 (25.3)	42 (32.3)	85 (65.3)	23 (17.6)

*Total (200)*	*100 (50)*	*90 (45)*	*190 (95)*	*140 (70)*	*40 (20)*	*80 (40)*	*100 (50)*	*50 (25)*	*130 (65)*	*170 (85)*	*40 (20)*	*50 (25)*	*50 (25)*	*60 (30)*	*120 (60)*	*30 (15)*

^*∗*^OX: oxytetracycline (30 *μ*g/disk), Deox: doxycycline (30 *μ*g/disk), Gen: gentamicin (10 *µ*g/disk), Amk: amikacin (30 u/disk), Nal: nalidixic acid (30 *μ*g/disk), En: enrofloxacin (5 *µ*g/disk), Cip: ciprofloxacin (5 *µ*g/disk), Amc: amoxicillin-clavulanic acid (30 *μ*g/disk), Amx: amoxicillin (10 *µ*g/disk); Amp: ampicillin (10 u/disk), Cf: cefazolin (30 *μ*g/disk), Cft: ceftiofur (30 *μ*g/disk), Ctz: ceftazidime (30 *μ*g/disk), Cfx: cefotaxime (30 *μ*g/disk), Sult: sulfamethoxazole-trimethoprim (25 *μ*g/disk), and C30: chloramphenicol (30 *µ*g/disk).

**Table 3:** Distribution of antibiotic resistance genes among the uropathogenic *E. coli* strains isolated from Iranian dogs.

Types of samples (number positive)	Antibiotic resistance genes (%)										
	*tetA*	*tetB*	*aac(3)-IV*	*qnr*	*CITM*	*blaSHV*	*aadA1*	*sul1*	*dfrA1*	*cat1*	*cmlA*
Healthy dogs											
Male (30)	17 (56.6)	10 (33.3)	28 (93.3)	25 (83.3)	23 (76.6)	20 (66.6)	10 (33.3)	17 (56.6)	16 (53.3)	3 (10)	1 (3.3)
Female (40)	8 (20)	6 (15)	20 (50)	15 (37.5)	14 (35)	10 (25)	5 (12.5)	12 (30)	13 (32.5)	1 (2.5)	—
Total (70)	25 (35.7)	16 (22.8)	48 (68.5)	40 (57.1)	37 (52.8)	30 (42.8)	15 (21.4)	29 (41.4)	29 (41.4)	4 (5.71)	1 (1.4)

Dogs with UTIs											
Male (70)	40 (57.1)	22 (31.4)	61 (87.1)	30 (42.8)	38 (54.2)	27 (38.5)	16 (22.8)	29 (41.4)	25 (35.7)	5 (7.1)	3 (4.2)
Female (60)	28 (46.6)	16 (26.6)	45 (75)	24 (40)	30 (50)	18 (30)	10 (16.6)	22 (36.6)	16 (26.6)	3 (5)	1 (1.6)
Total (130)	68 (52.3)	38 (29.2)	106 (81.5)	54 (41.5)	68 (52.3)	45 (34.6)	26 (20)	51 (39.2)	41 (31.5)	8 (6.15)	4 (3.0)

*Total (200)*	*93 (46.5)*	*54 (27)*	*154 (77)*	*94 (47)*	*105 (52.5)*	*75 (37.5)*	*41 (20.5)*	*80 (40)*	*70 (35)*	*12 (6)*	*5 (2.5)*

## References

[1] Hagman R., Greko C. (2005). Papers & Articles. *The Veterinary Record*.

[2] Cooke C. L., Singer R. S., Jang S. S., Hirsh D. C. (2002). Enrofloxacin resistance in Escherichia coli isolated from dogs with urinary tract infections. *Journal of the American Veterinary Medical Association*.

[3] Chang S.-K., Lo D.-Y., Wei H.-W., Kuo H.-C. (2015). Antimicrobial resistance of Escherichia coli isolates from canine urinary tract infections. *Journal of Veterinary Medical Science*.

[4] Wagner S., Gally D. L., Argyle S. A. (2014). Multidrug-resistant *Escherichia coli* from canine urinary tract infections tend to have commensal phylotypes, lower prevalence of virulence determinants and ampC-replicons. *Veterinary Microbiology*.

[5] Rzewuska M., Czopowicz M., Kizerwetter-Świda M., Chrobak D., Błaszczak B., Binek M. (2015). Multidrug resistance in Escherichia coli strains isolated from infections in dogs and cats in Poland (2007-2013). *The Scientific World Journal*.

[6] Momtaz H., Karimian A., Madani M. (2013). Uropathogenic *Escherichia coli* in Iran: serogroup distributions, virulence factors and antimicrobial resistance properties. *Annals of Clinical Microbiology and Antimicrobials*.

[7] Momtaz H., Farzan R., Rahimi E., Safarpoor Dehkordi F., Souod N. (2012). Molecular characterization of Shiga toxin-producing Escherichia coli isolated from ruminant and donkey raw milk samples and traditional dairy products in Iran. *The Scientific World Journal*.

[8] Momtaz H., Safarpoor Dehkordi F., Rahimi E., Ezadi H., Arab R. (2013). Incidence of Shiga toxin-producing Escherichia coli serogroups in ruminant's meat. *Meat Science*.

[9] Dehkordi F. S., Yazdani F., Mozafari J., Valizadeh Y. (2014). Virulence factors, serogroups and antimicrobial resistance properties of *Escherichia coli* strains in fermented dairy products. *BMC Research Notes*.

[10] Momtaz H., Dehkordi F. S., Hosseini M. J., Sarshar M., Heidari M. (2013). Serogroups, virulence genes and antibiotic resistance in Shiga toxin-producing *Escherichia coli*isolated from diarrheic and non-diarrheic pediatric patients in Iran. *Gut Pathogens*.

[11] Hemmatinezhad B., Khamesipour F., Mohammadi M., Safarpoor Dehkordi F., Mashak Z. (2015). Microbiological Investigation of O-Serogroups, Virulence Factors and Antimicrobial Resistance Properties of Shiga Toxin-Producing Escherichia Coli Isolated from Ostrich, Turkey and Quail Meats. *Journal of Food Safety*.

[12] Momtaz H., Safarpoor Dehkordi F., Taktaz T., Rezvani A., Yarali S. (2012). Shiga toxin-producing Escherichia coli isolated from bovine mastitic milk: Serogroups, virulence factors, and antibiotic resistance properties. *The Scientific World Journal*.

[13] Ranjbar R., Masoudimanesh M., Dehkordi F. S., Jonaidi-Jafari N., Rahimi E. (2017). Shiga (Vero)-toxin producing Escherichia coli isolated from the hospital foods; virulence factors, o-serogroups and antimicrobial resistance properties. *Antimicrobial Resistance & Infection Control*.

[14] Drazenovich N., Ling G. V., Foley J. (2004). Molecular investigation of Escherichia coli strains associated with apparently persistent urinary tract infection in dogs. *Journal of Veterinary Internal Medicine*.

[15] Windahl U., Holst B. S. T., Nyman A., Grönlund U., Bengtsson B. (2014). Characterisation of bacterial growth and antimicrobial susceptibility patterns in canine urinary tract infections. *BMC Veterinary Research*.

[16] Philip R. E., William E. (1986). *Identification ofEnterobacteriaceae by Biochemical Reactions*.

[17] Sambrook J., Russell D. (2001). *Molecular, cloning, a laboratory manual*.

[18] Wayne P. (2012). *Clinical and Laboratory Standards Institute (CLSI). Performance standards for antimicrobial susceptibility testing*.

[19] Sanchez S., Stevenson M. A. M., Hudson C. R. (2002). Characterization of multidrug-resistant Escherichia coli isolates associated with nosocomial infections in dogs. *Journal of Clinical Microbiology*.

[20] Ling G. V., Norris C. R., Franti C. E. (2001). Interrelations of organism prevalence, specimen collection method, and host age, sex, and breed among 8, 354 canine urinary tract infections (1969-1995). *Journal of Veterinary Internal Medicine*.

[21] Wong C., Epstein S. E., Westropp J. L. (2015). Antimicrobial susceptibility patterns in urinary tract infections in dogs (2010-2013). *Journal of Veterinary Internal Medicine*.

[22] Wynn S. G., Witzel A. L., Bartges J. W., Moyers T. S., Kirk C. A. (2016). Prevalence of asymptomatic urinary tract infections in morbidly obese dogs. *PeerJ*.

[23] Weese J. S., Blondeau J. M., Boothe D. (2011). Antimicrobial use guidelines for treatment of urinary tract disease in dogs and cats: antimicrobial guidelines working group of the international society for companion animal infectious diseases. *Veterinary Medicine international*.

[24] Robicsek A., Strahilevitz J., Jacoby G. A. (2006). Fluoroquinolone-modifying enzyme: a new adaptation of a common aminoglycoside acetyltransferase. *Nature Medicine*.

[25] Yamane K., Wachino J.-I., Suzuki S. (2007). New plasmid-mediated fluoroquinolone efflux pump, QepA, found in an Escherichia coli clinical isolate. *Antimicrobial Agents and Chemotherapy*.

[26] Briñas L., Zarazaga M., Sáenz Y., Ruiz-Larrea F., Torres C. (2002). *β*-Lactamases in ampicillin-resistant *Escherichia coli* isolates from foods, humans, and healthy animals. *Antimicrobial Agents and Chemotherapy*.

[27] Williamson N. L., Frank L. A., Hnilica K. A. (2002). Effects of short-term trimethoprim-sulfamethoxazole administration on thyroid function in dogs. *Journal of the American Veterinary Medical Association*.

[28] Spohr A., Schjøth B., Wiinberg B. *Antibiotic Use Guidelines for Companion Animal Practice. A guide to achieving the best possible clinical response with the lowest risk of antibiotic resistance*.

[29] Westropp J. L., Sykes J. E., Irom S. (2012). Evaluation of the Efficacy and Safety of High Dose Short Duration Enrofloxacin Treatment Regimen for Uncomplicated Urinary Tract Infections in Dogs. *Journal of Veterinary Internal Medicine*.

[30] SafarpoorDehkordi F., Gandomi H., AkhondzadehBasti A., Misaghi A., Rahimi E. (2017). Phenotypic and genotypic characterization of antibiotic resistance of methicillin resistant from hospital food. *Antimicrobial Resistance & Infection Control*.

[31] Atapoor S., Safarpoor Dehkordi F., Rahimi E. (2014). Detection of Helicobacter pylori in various types of vegetables and salads. *Jundishapur Journal of Microbiology*.

[32] Momtaz H., Dehkordi F. S., Rahimi E., Asgarifar A., Momeni M. (2013). Virulence genes and antimicrobial resistance profiles of Staphylococcus aureus isolated from chicken meat in Isfahan province, Iran. *Journal of Applied Poultry Research*.

[33] Hasanpour Dehkordi A., Khaji L., Sakhaei Shahreza M. H. (2017). One-year prevalence of antimicrobial susceptibility pattern of methicillin-resistant Staphylococcus aureus recovered from raw meat. *Tropical Biomedicine*.

[34] Ghorbani F., Gheisari E., Dehkordi F. S. (2016). Genotyping of vacA alleles of Helicobacter pylori strains recovered from some Iranian food items. *Tropical Journal of Pharmaceutical Research*.

[35] Safarpoor Dehkordi F., Parsaei P., Saberian S. (2012). Prevalence study of theileria annulata by comparison of four diagnostic techniques in Southwest Iran. *Bulgarian Journal of Veterinary Medicine*.

[36] Safarpoor Dehkordi F., Barati S., Momtaz H., Hosseini Ahari S. N., Nejat Dehkordi S. (2013). Comparison of shedding, and antibiotic resistance properties of listeria monocytogenes isolated from milk, feces, urine, and vaginal secretion of bovine, ovine, caprine, buffalo, and camel species in Iran. *Jundishapur Journal of Microbiology*.

[37] Rahimi E., Sepehri S., Safarpoor Dehkordi F., Shaygan S., Momtaz H. (2014). Prevalence of Yersinia species in traditional and commercial dairy products in Isfahan Province, Iran. *Jundishapur Journal of Microbiology*.

[38] Momtaz H., Davood Rahimian M., Safarpoor Dehkordi F. (2013). Identification and characterization of Yersinia enterocolitica isolated from raw chicken meat based on molecular and biological techniques. *Journal of Applied Poultry Research*.

[39] Momtaz H., Dehkordi F. S., Rahimi E., Asgarifar A. (2013). Detection of Escherichia coli, Salmonella species, and Vibrio cholerae in tap water and bottled drinking water in Isfahan, Iran. *BMC Public Health*.

[40] Shahrani M., Dehkordi F. S., Momtaz H. (2014). Characterization of Escherichia coli virulence genes, pathotypes and antibiotic resistance properties in diarrheic calves in Iran. *Biological Research*.

[41] Safarpoor Dehkordi F., Haghighi N., Momtaz H., Salari Rafsanjani M., Momeni M. (2013). Conventional Vs real-time PCR for detection of bovine herpes virus type 1 in aborted bovine, buffalo and camel Foetuses. *Bulgarian Journal of Veterinary Medicine*.

[42] Mousavi S., Dehkordi F., Rahimi E. (2014). Virulence factors and antibiotic resistance of Helicobacter pylori isolated from raw milk and unpasteurized dairy products in Iran. *Journal of Venomous Animals and Toxins including Tropical Diseases*.

[43] Madahi H., Rostami F., Rahimi E., Dehkordi F. S. (2014). Prevalence of enterotoxigenic Staphylococcus aureus isolated from chicken nugget in Iran. *Jundishapur Journal of Microbiology*.

[44] Dehkordi F. S., Khamesipour F., Momeni M. (2014). Brucella abortus and Brucella melitensis in Iranian bovine and buffalo semen samples: The first clinical trial on seasonal, Senile and geographical distribution using culture, Conventional and real-time polymerase chain reaction assays. *Kafkas Universitesi Veteriner Fakultesi Dergisi*.

[45] Dehkordi F. S., Haghighi Borujeni M. R., Rahimi E., Abdizadeh R. (2013). Detection of toxoplasma gondii in raw caprine, ovine, buffalo, bovine, and camel milk using cell cultivation, cat bioassay, capture ELISA, and PCR methods in Iran. *Foodborne Pathogens and Disease*.

[46] Rahimi E., Yazdanpour S., Dehkordi F. S. (2013). Detection of toxoplasma gondii antibodies in various poultry meat samples using enzyme linked immuno sorbent assay and its confirmation by polymerase chain reaction. *Journal of Pure and Applied Microbiology*.

[47] Safarpoor Dehkordi F., Valizadeh Y., Birgani T. A., Dehkordi K. G. (2013). Prevalence Study of Brucella melitensis and Brucella abortus in cow's milk using dot enzyme linked immuno sorbent assay and duplex polymerase chain reaction. *Journal of Pure and Applied Microbiology*.

[48] Nejat S., Momtaz H., Yadegari M., Nejat S., Safarpour Dehkordi F., Khamesipour F. (2015). Seasonal, geographical, age and breed distributions of equine viral arteritis in Iran. *Kafkas Universitesi Veteriner Fakultesi Dergisi*.

[49] Dormanesh B., Safarpoor Dehkordi F., Momtaz H. (2014). Virulence factors and O-Serogroups profiles of uropathogenic Escherichia coli isolated from Iranian pediatric patients. *Iranian Red Crescent Medical Journal*.

